# Application of metagenomic next-generation sequencing in HIV-negative hematogenous disseminated tuberculosis

**DOI:** 10.3389/fcimb.2026.1851741

**Published:** 2026-07-02

**Authors:** Lingxin Luo, Jvrong Zhan, Zhen Wang, Xianzhi Du, Na Li

**Affiliations:** Department of Respiratory and Critical Care Medicine, The Second Affiliated Hospital of Chongqing Medical University, Chongqing, China

**Keywords:** diagnosis, hematogenous disseminated tuberculosis, HIV-negative, metagenomic next-generation sequencing, tuberculosis

## Abstract

**Background:**

Hematogenous disseminated tuberculosis (Hematogenous disseminated tuberculosis, HDTB) is a rare, critical form of tuberculosis with a high case fatality ratio and is uncommon in HIV-negative patients. Early recognition of this disease is difficult, and limitations of traditional testing methods often lead to delayed diagnosis. This study aims to investigate the value of metagenomic Next-Generation Sequencing (metagenomic Next-Generation Sequencing, mNGS), as a promising tool, in the diagnosis of hematogenous disseminated tuberculosis in HIV-negative (Human Immunodeficiency Virus, HIV) patients.

**Methods:**

A retrospective analysis was conducted of the clinical data of 10 HIV-negative patients with hematogenous disseminated tuberculosis confirmed by mNGS.

**Results:**

All patients had pre-existing diseases that could lead to impaired immune function. Common symptoms included hyperpyrexia, cough, and dyspnea, and 6 patients developed respiratory failure. C-reactive protein (C-reactive protein, CRP) and procalcitonin (procalcitonin, PCT) levels were both elevated, and PCT was markedly elevated in more than half of the patients, using 0.5 ng/mL as the cutoff value. Most patients had markedly elevated D-dimer levels accompanied by thrombotic events, including 3 patients with concomitant pulmonary embolism. Chest imaging showed patchy pulmonary opacities, and 2 patients had atypical bilateral pleural effusion; these nonspecific findings were easily confused with those of other diseases. Blood mNGS detected Mycobacterium tuberculosis within 2 to 3 days. According to the presence or absence of concomitant pulmonary tuberculosis, the patients were divided into the pulmonary tuberculosis subgroup (pulmonary tuberculosis subgroup, PTB) and the non-pulmonary tuberculosis subgroup (non-pulmonary tuberculosis subgroup, non-PTB). The oxygenation index was significantly lower in the pulmonary tuberculosis subgroup than in the non-pulmonary tuberculosis subgroup (P = 0.037). All cases of pulmonary embolism occurred in the pulmonary tuberculosis subgroup, but the difference was not statistically significant.

**Conclusions:**

HIV-negative patients with hematogenously disseminated tuberculosis have atypical clinical manifestations and are prone to incorrect diagnosis. The application of mNGS helps shorten diagnostic delays and accelerate disease control, providing an effective supplementary diagnostic pathway when conventional testing methods cannot identify the pathogen.

## 1 Introduction

Tuberculosis is a respiratory-transmitted infectious disease caused by Mycobacterium tuberculosis (M. tuberculosis). It can involve multiple organs throughout the body, causing pulmonary and extrapulmonary infection. According to data from the WHO Global Tuberculosis Report 2025, there were 10.7 million new tuberculosis cases worldwide in 2024, with 1.23 million related deaths during the year, and tuberculosis remains the single infectious disease causing the highest number of deaths (World Health Organization, 2025). Mycobacterium tuberculosis can disseminate through the blood or lymph. Disseminated tuberculosis can be diagnosed if Mycobacterium tuberculosis is isolated from the blood or detected in two or more noncontiguous organs (Iseman, 2000). The geographic distribution of cases varies substantially, and China is a high-burden region for tuberculosis. Problems such as multidrug-resistant tuberculosis and infection complicated by pre-existing diseases continue to increase the pressure on disease prevention and control. Disseminated tuberculosis is relatively uncommon, accounting for 1% to 5% of all tuberculosis cases. As a form of disseminated tuberculosis, hematogenous disseminated pulmonary tuberculosis has an insidious onset, progresses rapidly, and is difficult to diagnose, which can readily delay clinical diagnosis and treatment (Ryndak et al., 2014). Its clinical manifestations are usually nonspecific, and diagnosis requires bacteriologic or molecular biologic evidence (Krishnan et al., 2010; Sharma et al., 2016). Because early recognition and diagnosis are difficult, treatment of hematogenous disseminated tuberculosis is often delayed (Tyrrell et al., 2012). Current global progress in tuberculosis prevention and control has still not met the anticipated strategic targets, and conventional detection methods have limitations. mNGS is a novel, culture-independent, high-throughput sequencing technology that has been increasingly applied to etiologic diagnosis of infectious diseases across organ systems (Ramachandran and Wilson, 2020; Diao et al., 2021; Chen et al., 2022), with particularly notable advantages in diagnostically challenging and critically ill cases (Qian et al., 2021; Chen et al., 2022). To date, studies have suggested that mNGS has potential clinical utility in the diagnosis of pulmonary and extrapulmonary tuberculosis, but reports on its application in disseminated tuberculosis remain limited (Yan et al., 2020; Huang et al., 2023). By describing the clinical characteristics of 10 HIV-negative patients with hematogenous disseminated tuberculosis confirmed by mNGS, this study indicates that mNGS has clinical utility in these selected cases.

## 2 Materials and methods

### 2.1 Study design

This retrospective study included 10 HIV-negative patients with hematogenous disseminated tuberculosis confirmed by mNGS at the Second Affiliated Hospital of Chongqing Medical University from February 2021 to January 2023. Patient medical records were extracted from the inpatient system, including baseline information, medical history, examination results, treatment plans, and prognosis. Patients were categorized into a pulmonary tuberculosis subgroup and a non-pulmonary tuberculosis subgroup according to the presence of concomitant pulmonary tuberculosis, and differences between the two groups were compared. This study was approved by the Ethics Committee of the Second Affiliated Hospital of Chongqing Medical University, with a waiver of informed consent for anonymous data collection.

### 2.2 Study methods

#### 2.2.1 Diagnostic criteria for hematogenously disseminated tuberculosis

Disseminated tuberculosis can be diagnosed if any of the following criteria are met (Krishnan et al., 2010; Sharma et al., 2016; Khan et al., 2016; Jolobe, 2017): (1) Mycobacterium tuberculosis is isolated from blood, bone marrow, liver biopsy tissue, or at least two noncontiguous organs, or molecular biological testing for tuberculosis is positive; (2) Mycobacterium tuberculosis is isolated from one organ, and caseating granuloma is histologically confirmed in bone marrow, liver biopsy tissue, or another noncontiguous site; (3) caseating granuloma is histologically identified in one organ, and imaging findings are consistent with miliary tuberculosis.

#### 2.2.2 mNGS testing procedures

Sequencing platform description: The sequencing platform used in this study was the Illumina NextSeq 550Dx (San Diego, CA, USA) (Jeon et al., 2014; Zhang et al., 2019). Library construction was performed according to the BGISeq-500 standard workflow, a combination that has been validated as feasible in a clinical laboratory, and DNA libraries were assessed for quality and quality control using an Agilent 2100 Bioanalyzer. Sample processing and DNA extraction: Qualified clinical samples, including blood, sputum, pleural effusion, and cerebrospinal fluid, were collected in accordance with aseptic operating procedures. Control system and quality control description: A molecular tag named UMSI, a quality control nucleotide sequence, was added to the samples as an internal reference, and sample contamination was evaluated together with an external negative control, a blank control tested in parallel with the clinical samples. Specific operating instructions: Sample DNA was extracted using the TIANamp Micro DNA Kit from Tiangen Biotech (Beijing) Co. Ltd. according to the manufacturer’s instructions. Following the standard workflow of the BGISeq-500 sequencing platform, DNA libraries were constructed through DNA fragmentation, end ligation, and PCR amplification. After removing low-quality, low-complexity, and short-sequence reads, human host sequences were aligned to the human reference genomes (hg19, hg38, and YH sequences) using Burrows-Wheeler alignment (BWA). The remaining microbial sequences were simultaneously aligned against and classified using four major microbial genome databases containing bacteria, viruses, fungi, and parasites. The databases were downloaded from and optimized based on public databases, including the National Center for Biotechnology Information (NCBI) and the China National GeneBank DataBase (CNGBdb). Contamination assessment: An external negative control and clinical samples were tested in parallel. Microorganisms detected in the negative control, such as common environmental bacteria, were not reported as pathogenic pathogens if they were present in clinical samples but the read count was not significantly higher than that in the negative control.

#### 2.2.3 Interpretation of mNGS results

Considering that Mycobacterium tuberculosis is intracellular bacteria and that its cell wall is difficult to lyse, DNA extraction is relatively difficult. Detection of Mycobacterium tuberculosis can be determined when the following conditions are met: (1) its standardized stringently mapped read number (SDSMRN) ranks among the top 20; (2) it ranks first within its genus; and (3) SDSMRN ≥ 1 (Qian et al., 2021). Even when only 1 specific sequence is detected, its pathogenicity should be highly suspected, and the specific determination should be made based on comprehensive clinical judgment (Langelier et al., 2018).

#### 2.2.4 Statistical methods

IBM SPSS 26.0 software was used to compare differences between the two groups. After normality testing, normally distributed data were analyzed using the independent-samples t test, nonnormally distributed data were analyzed using the independent-samples nonparametric test, and count data were compared using Fisher’s exact test. P < 0.05 was considered statistically significant.

## 3 Results

### 3.1 General patient characteristics

Among the 10 patients, 7 were male (70%) and 3 were female (30%). The mean age of the patients was 61 years (range, 29 to 81 years). All patients had at least one pre-existing disease. Among them, 2 patients had a recent history of immunosuppressive drug use, 3 patients had a long-term history of smoking and alcohol use, 2 patients had a long-term history of dust exposure, and 1 patient was pregnant and experienced intrauterine fetal death of her infant (**Table 1**).

**Table 1:** General patient characteristics.

Patient no.	Male or female	Age (average [range] [yrs])	Underlying disease(s)	Immunosuppressive therapy (drug)	Smoker	Drinker	Dust exposure	Durations from onset of illness until hospital admission
1	M	80	Rheumatic heart disease, cirrhosis	No	Yes	Yes	No	89 days
2	M	77	Chronic bronchitis, Hypertension, diabetes, Second-degree atrioventricular block, With a pacemaker	No	No	No	No	More than 2 years
3	M	69	Lung cancer	No	Yes	Yes	Yes	30 days
4	F	38	Aplastic anemia	Yes (tacrolimus)	No	No	No	6 days
5	M	45	Diabetes, hypertension, Kidney transplant status, Rheumatic heart disease	Yes (unknown drug)	No	No	No	60 days
6	F	29	Pregnancy, intrauterine fetal death	No	No	No	No	3 days
7	M	59	Elevated white blood cells, Hypertension	No	No	No	No	3 days
8	M	66	Hypertension, Coronary heart disease	No	Yes	Yes	Yes	7 days
9	F	81	Coronary heart disease, Atrial fibrillation	No	No	No	No	30 days
10	M	66	Diabetes, drug-induced liver injury	No	No	No	No	10 days
Total	7M, 3F	61(29-81)	10/10	2/10	3/10	3/10	2/10	

Unless otherwise indicated, the data in the “Total” row represents the proportion of patients. M, male; F, female.

### 3.2 Clinical symptoms

The patients mainly presented with general symptoms: hyperpyrexia (>39 °C, 100%) and fatigue (40%); and respiratory symptoms: cough (80%), expectoration (50%), and dyspnea (70%) (**Table 2**).

**Table 2:** Clinical manifestation.

Patient no.	Body temperature > 39°C	Weakness	Cough	Expectoration	Dyspnea	Chest pain	Palpitation	Dizziness	Headache	Weight loss
1	Yes	No	Yes	Yes	No	No	No	Yes	No	No
2	Yes	Yes	Yes	Yes	Yes	No	Yes	No	No	No
3	Yes	No	Yes	Yes	No	No	No	No	No	No
4	Yes	No	Yes	Yes	No	No	No	No	No	No
5	Yes	Yes	Yes	No	Yes	No	No	No	Yes	Yes
6	Yes	Yes	No	No	Yes	No	No	No	No	Yes
7	Yes	No	Yes	Yes	Yes	Yes	Yes	No	No	No
8	Yes	No	No	No	Yes	No	No	No	No	No
9	Yes	No	Yes	No	Yes	No	No	No	No	No
10	Yes	Yes	Yes	No	Yes	No	No	Yes	No	No
Total	10/10	4/10	8/10	5/10	7/10	1/10	2/10	2/10	1/10	2/10

The data in the "Total" row represents the proportion of patients.

### 3.3 Laboratory examinations

Two-thirds of the patients developed respiratory failure. CRP and PCT were elevated in all patients, and PCT was significantly elevated in 60% of patients, with 0.5 ng/mL used as the cutoff value. Lymphocyte counts were decreased in 80% of patients. CD4+ T-cell and CD8+ T-cell counts were measured in 4 patients, and all were decreased. In most patients, interleukin-2 receptor (IL-2R), interleukin-6 (IL-6), interleukin-8 (IL-8), interleukin-10 (IL-10), tumor necrosis factor-α (TNF-α), and interferon-γ (IFN-γ) were elevated to varying degrees. D-dimer was measured in 9 patients, and the levels were elevated in all of them, with marked elevation in 7 patients (**Table 3**).

**Table 3:** Laboratory examinations.

Items (Normal range[unit])	P 1	P 2	P 3	P 4	P 5	P 6	P 7	P 8	P 9	P 10
Oxygenation index (400.00-500.00mmHg)	481.00	310.00	227.00	–	385.00	152.00	256.00	282.00	189.00	159.00
Leukocyte count (3.50-9.50×10^9/L)	3.23	4.48	12.36	5.12	14.60	7.70	20.18	11.22	7.64	4.65
Neutrophil percentage (45.00-75.00%)	80.20	79.00	90.50	76.40	81.20	92.60	–	91.30	97.0	88.80
Lymphocyte percentage (20.00-50.00%)	9.10	12.70	4.80	13.90	10.20	5.90	–	5.80	1.80	8.90
Neutrophil count (1.80-6.30×10^9/L)	2.60	3.54	11.19	3.92	11.86	7.14	–	10.24	7.41	4.13
Lymphocyte count (1.10-3.20×10^9/L)	0.29	0.57	0.59	0.71	1.50	0.46	–	0.65	0.14	0.41
Hemoglobin (115.00-150.00g/L)	77.00	121.00	76.00	87.00	91.00	88.00	60.00	139.00	101.00	116.00
Platelet count (100.00-300.00×10^9/L)	52.00	191.00	334.00	167.00	336.00	40.00	84.00	274.00	216.00	179.00
C-reactive protein (<10.00mg/L)	154.78	36.47	>200.00	110.42	44.59	142.01	>200.00	77.91	>200.00	124.24
Procalcitonin (0-0.05ng/ml)	2.66	0.13	0.24	0.21	0.35	15.88	14.17	0.88	0.82	2.34
Albumin (40.00-55.00g/L)	32.30	31.90	32.10	36.00	48.10	24.90	28.10	29.00	21.10	28.50
Alanine aminotransferase (9.00-50.00U/L)	7.00	25.00	31.00	13.00	345.00	490.00	22.00	65.00	164.00	278.00
Aspartate aminotransferase (15.00-40.00U/L)	28.00	28.00	20.00	55.00	170.00	4825.00	39.00	47.00	282.00	331.00
Alkaline phosphatase (45.00-125.00U/L)	96.00	103.00	135.00	86.00	137.00	66.00	53.00	86.00	149.00	634.00
Gamma-glutamyltransferase (10.00-60.00U/L)	71.00	97.00	133.00	52.00	129.00	23.00	53.00	62.00	111.00	784.00
D-dimer (0-550.00ng/ml)	4058.30	803.00	2916.20	–	736.00	5862.40	>10000.00	6624.60	3882.40	3613.10
Interleukin-2 receptor (223.00- 710.00U/ml)	–	–	–	996.00	–	1932.00	2696.08	2679.00	3337.00	>7500.00
Interleukin-6 (0-5.90pg/ml)	155.79	–	–	60.01	–	242.00	584.08	21.70	172.00	380.00
Interleukin-8 (0-62.00pg/ml)	–	–	–	27.30	–	289.00	381.10	198.00	215.00	415.00
Interleukin-10 (0-9.10pg/ml)	9.48	–	–	7.37	–	28.20	15.27	5.22	5.37	26.00
Tumor necrosis factor-alpha (0-8.10pg/ml)	1.96	–	–	2.02	–	45.90	1.46	23.40	20.80	184.00
Interferon-gamma (0-7.42pg/ml)	69.55	–	–	27.89	–	–	4.59	–	–	–
CD4(+) T cell counts (500.00-1440.00/uL)	–	–	205.00	–	–	130.00	–	–	216.00	164.00
CD8(+) T cell counts (238.00-1250.00/uL)	–	–	216.00	–	–	140.00	–	–	172.00	224.00
CD4/CD8 (1.00-2.47)	–	–	0.95	–	–	0.93	–	–	1.26	0.73
HIV antibody	Negative	Negative	Negative	Negative	Negative	Negative	Negative	Negative	Negative	Negative

The “P” represents the patient.

### 3.4 Imaging examinations

**Figure 1:**
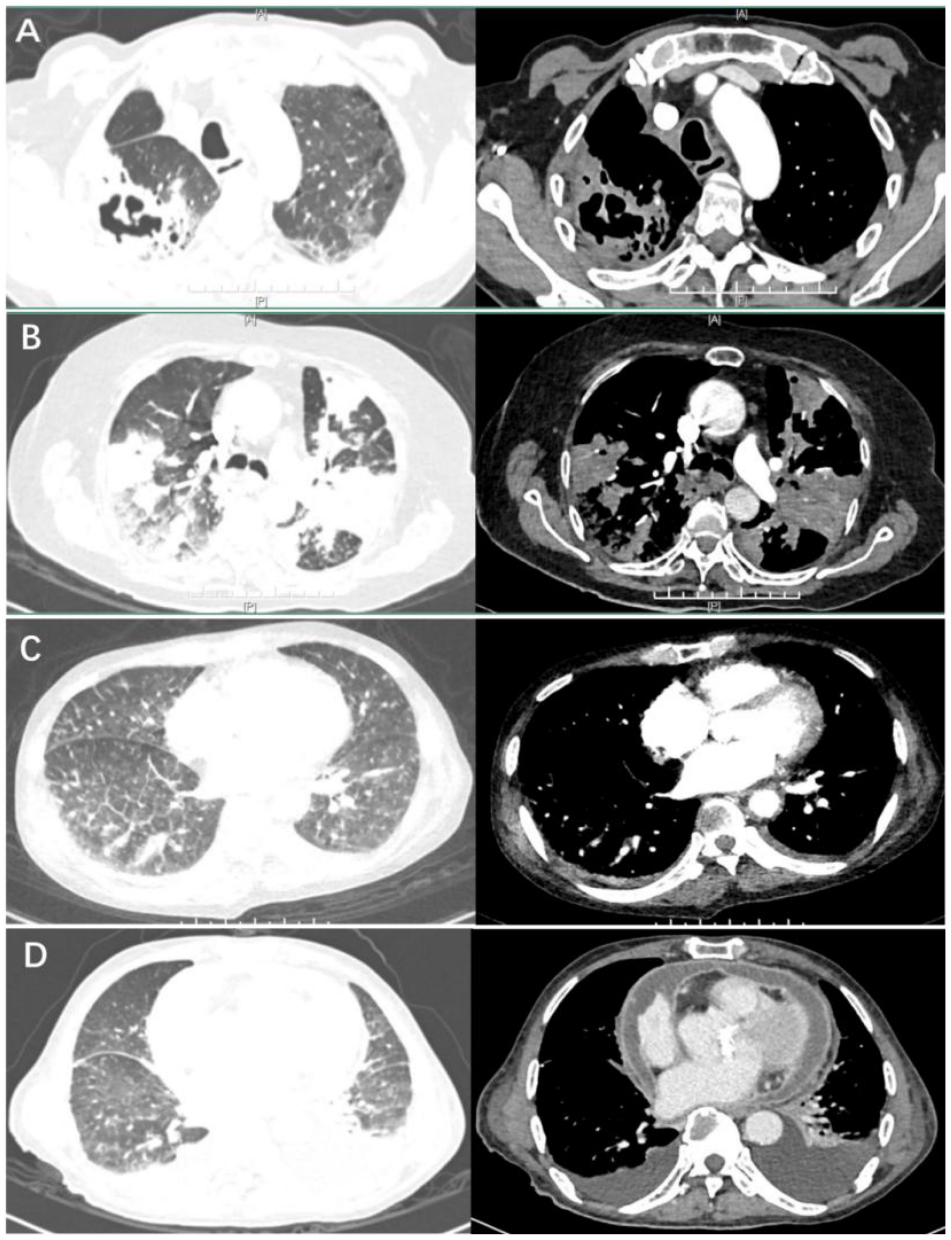
**(A)** (Patient 3), lung window (left) and mediastinal window (right): Multiple patchy subpleural ground-glass opacities are seen in both lungs, with indistinct margins; patchy consolidation is seen in the posterior segment of the right lower lobe, with air bronchograms. Irregular cavitation is seen within the area of consolidation in the posterior segment of the right lower lobe, with uneven wall thickness and no obvious air-fluid level. **(B)** (Patient 9), lung window (left) and mediastinal window (right): Diffusely distributed scattered punctate and patchy high-density opacities are seen in both lungs, with some lesions coalescing into small patchy areas of consolidation, most prominent in the middle and lower zones of both lungs. The lesions are predominantly pulmonary parenchymal infiltrates. No obvious enlarged mediastinal lymph nodes or pleural effusion is seen. **(C)** (Patient 7), lung window (left) and mediastinal window (right): Diffusely distributed micronodular opacities are seen in both lungs, distributed along the bronchovascular bundles and interlobular septa, accompanied by nodular thickening of the interlobular septa, showing typical miliary changes. Diffuse small intrapulmonary nodules are clearly visualized, the mediastinal structures are clear, and there is no pleural thickening or effusion. **(D)** (Patient 8), lung window (left) and mediastinal window (right): Scattered nodular opacities and patchy hazy opacities are distributed in both lungs, with lesions extensively involving all lobes bilaterally. Bilateral pleural effusion with a small amount of pericardial effusion is observed. The pulmonary nodules and patchy opacities have relatively well-defined margins, without cavitation or obvious calcification.

All 10 patients underwent imaging examinations, including chest CT, brain MRI, CT pulmonary angiography, brain CT, and lower extremity venous ultrasound. All patients had pulmonary lesions, which appeared as patchy or spotted high-density opacities, ground-glass opacities, or consolidations of varying degrees; 9 patients (90%) had bilateral lung involvement. Patients 3 and 9 had pulmonary cavities, patients 1 and 8 had bilateral pleural effusion, and patients 7 and 8 had diffuse lung nodules (**Figure 1**). Patients 6 and 8 had concomitant brain infarction, patients 2, 3, and 9 had concomitant pulmonary embolism, and patients 3 and 7 had concomitant lower extremity venous thrombosis.

### 3.5 Conventional microbiological examination

Blood cultures were negative in all patients. Sputum acid-fast bacillus smear testing was performed in 6 patients, and all results were negative. Sputum culture was performed in 6 patients; multidrug-resistant Acinetobacter baumannii was detected in patient 2, and yeast-like fungi were detected in patient 9. Patients 1 and 8 underwent pleural puncture: routine biochemical analysis of the pleural effusion in patient 1 showed a nucleated cell count of 500 × 10^6^/L, predominantly polymorphonuclear cells, protein 30 g/L, LDH >200 U/L, normal ADA, and a negative pleural effusion culture. Patient 5 underwent lumbar puncture, and routine biochemical analysis of the cerebrospinal fluid showed a nucleated cell count of >500 × 10^6^/L, predominantly polymorphonuclear cells, and elevated protein; both cerebrospinal fluid smear and culture were negative (**Table 4**).

**Table 4:** Conventional microbiological examination.

Items (unit)	Patient 1	Patient 2	Patient 3	Patient 4	Patient 5	Patient 6	Patient 7	Patient 8	Patient 9	Patient 10
G test (pg/ml)	45.30	<37.50	223.00	<37.50	52.20	40.40	<37.50	<37.50	<37.50	<37.50
GM test (S/CO)	<0.25	<0.25	0.40	<0.25	<0.25	<0.25	0.30	<0.25	<0.25	0.40
TB-Ab IgG	–	–	Negative	–	–	–	–	Positive	Negative	–
TB-Ab IgM	–	–	Positive	–	–	–	–	Negative	Negative	–
T-SOPT	Positive	–	Negative	–	Negative	–	–	Negative	Negative	Negative
PPDa	Negative	–	++	Negative	–	–	–	Negative	–	–
Sputum smear	–	Negative	–	–	Negative	Negative	–	Negative	Negative	Negative
Sputum culture	–	Multidrug-resistant Acinetobacter baumannii	Negative	–	Negative	Negative	–	Negative	Yeaster-like fungus	–
Blood culture	Negative	Negative	Negative	Negative	Negative	Negative	Negative	Negative	Negative	Negative
Pleural fluid culture	–	–	–	–	–	–	–	Negative	–	–
Cerebrospinal fluid smear	–	–	–	–	Negative	–	–	–	–	–
Cerebrospinal fluid culture	–	–	–	–	Negative	–	–	–	–	–

G test, (1-3)-β-D-glucan test;GM test, galactomannan test;TB-Ab IgG, human tuberculosis antibody IgG test;TB-Ab IgM, human tuberculosis antibody IgM test;T-SOPT, interferon gamma release assay;PPD, purified protein derivative test for tuberculosis; smear, acid-fast staining for M. tuberculosis.

a: Reaction was observed between 48–72 hours for maximum induration size: 0-5mm is negative, 5-10mm is mildly positive +, 10-20mm is moderately positive ++, and over 20mm is strongly positive +++.

### 3.6 mNGS and other molecular biology test results

In this study, all 10 HIV-negative patients with negative conventional etiologic test results underwent mNGS testing and were diagnosed with hematogenous disseminated tuberculosis within 48 to 72 hours after testing. Subsequently, patients 2, 4, and 9 had positive Xpert MTB results in bronchoalveolar lavage fluid, patient 3 had a positive TB-DNA result in bronchoalveolar lavage fluid, and acid-fast bacilli were detected on multiple sputum acid-fast bacillus smears in patient 10 (**Table 5**).

**Table 5:** Results of mNGS and PCR.

Patient no.	Platform	Sample type(s)	Detected pathogen(s) (no. of species-specific reads)	PCR Results (Items)
1	NextSeq 550Dx Sequencing platform	Blood	Mycobacterium tuberculosis complex (5), Human herpesvirus 4 (5)	–
		Pleural fluid	Mycobacterium tuberculosis complex (1), Human herpesvirus 4 (3)	–
2	NextSeq 550Dx sequencing platform	Blood	Mycobacterium tuberculosis complex (1), Human herpesvirus 1 (2)	–
		BALF	–	Positive (Xpert MTB/RIF)
3	NextSeq 550Dx sequencing platform	Blood	Mycobacterium tuberculosis complex (9), Human herpesvirus 4 (3), Human herpesvirus 5 (1)	–
		BALF	–	Positive (TB-DNA)
4	NextSeq 550Dx sequencing platform	Blood	Mycobacterium tuberculosis complex (1)	–
		BALF	–	Positive (Xpert MTB/RIF)
5	NextSeq 550Dx sequencing platform	Blood	Mycobacterium tuberculosis complex (6), Human polyomavirus 1 (1), Human polyomavirus 2 (1)	–
		Cerebrospinal fluid	Mycobacterium tuberculosis complex (3), Human herpesvirus 4 (1)	–
6	NextSeq 550Dx sequencing platform	Blood	Mycobacterium tuberculosis complex (5)	–
		Sputum	Mycobacterium tuberculosis complex (214), Aspergillus flavus (4)	–
7	NextSeq 550Dx sequencing platform	Blood	Mycobacterium tuberculosis complex (1)	–
8	NextSeq 550Dx sequencing platform	Blood	Mycobacterium tuberculosis complex (2), Human herpesvirus 5 (4), propionibacterium acnes (34), Staphylococcus hominis (10), corynebacterium resistens (4), Moraxella osloensis (10)	–
		Pleural fluid	Mycobacterium tuberculosis complex (17), Staphylococcus hominis (3)	–
9	NextSeq 550Dx sequencing platform	Blood	Mycobacterium tuberculosis complex (54), Human herpesvirus 5 (11)	–
		BALF	–	Positive (Xpert MTB/RIF)
10	NextSeq 550Dx sequencing platform	Blood	Mycobacterium tuberculosis complex (16), Human herpesvirus 4 (4)	–

BALF, bronchoalveolar lavage fluid.

### 3.7 Final diagnosis

The diagnosis of each patient was comprehensively determined based on the diagnostic criteria for disseminated tuberculosis, including bacteriologic or molecular biologic evidence and imaging findings, together with the mNGS results and clinical manifestations. Final diagnoses were confirmed as follows: 10 cases of disseminated tuberculosis, including 2 complicated by tuberculous pleurisy, 1 complicated by tuberculous meningitis, 6 complicated by pulmonary tuberculosis, and 1 case of tuberculosis infection of unknown origin confirmed by blood testing alone.

### 3.8 Treatment and prognosis

Eight patients received anti-tuberculosis treatment, and 2 patients abandoned treatment. Among the 8 treated patients, 5 were discharged after clinical improvement, 1 died of multiple organ failure, 1 discontinued treatment, and 1 stopped medication because of hepatic function abnormality. Because the standard anti-tuberculosis treatment course is long and the loss-to-follow-up rate is high, the long-term prognosis remains unclear.

### 3.9 Subgroup analysis

Patients were divided into the pulmonary tuberculosis group (n=6) and the non-pulmonary tuberculosis group (n=4; 2 cases of tuberculous pleurisy, 1 case of tuberculous meningitis, and 1 case of tuberculosis of unknown cause). The oxygenation index was significantly lower in the pulmonary tuberculosis group (P = 0.037<0.05), and all pulmonary embolism events occurred in the pulmonary tuberculosis group, with no statistically significant difference (**Table 6**).

**Table 6:** Comparison of pulmonary tuberculosis subgroup and non-pulmonary tuberculosis subgroup.

Items (unit)	Pulmonary tuberculosis subgroup (n=6)	Non-pulmonary tuberculosis subgroup (n=4)	P-value
Age (yrs)	60.00 ± 21.41	62.50 ± 14.57	0.845
Gender			0.200
Male	3 (50%)	4 (100%)	
Female	3 (50%)	0 (0%)	
**Oxygenation index (mmHg)**	207.40 ± 64.54	351.00 ± 103.02	0.037
**Leukocyte count (×10^9^/L)**	6.99 ± 3.00	12.31 ± 7.09	0.135
**Neutrophil percentage (%)**	87.38 ± 8.03	84.23 ± 6.14	0.573
**Lymphocyte percentage (%)**	8.00 ± 4.71	8.37 ± 2.29	0.904
**Neutrophil count (×10^9^/L)**	6.22 ± 2.96	8.23 ± 4.95	0.460
**Lymphocyte count (×10^9^/L)**	0.48 ± 0.20	0.81 ± 0.62	0.453
**Hemoglobin (g/L)**	98.17 ± 17.7	91.75 ± 33.95	0.702
**Platelet count (×10^9^/L)**	187.83 ± 94.38	186.50 ± 139.77	0.986
**C-reactive protein (mg/L)**	135.52 ± 61.52	119.32 ± 70.87	0.710
**Procalcitonin (ng/ml)**	0.53 (0.19-5.73)	1.77 (0.48-11.29)	0.286
**Albumin (g/L)**	29.08 ± 5.41	34.38 ± 9.33	0.284
**Alanine aminotransferase (U/L)**	97.50(22.00-331.00)	43.50(10.75-275.00)	0.522
**Aspartate aminotransferase (U/L)**	168.50(26.00-1454.50)	43.00(30.75-139.25)	0.454
**Alkaline phosphatase (U/L)**	119.00 (81.00-270.25)	91.00 (61.25-126.75)	0.336
**Gamma-glutamyltransferase (U/L)**	104.00 (44.75-295.75)	66.50 (55.25-114.50)	0.670
**D-dimer (ng/ml)**	3415.42 ± 1824.75	5354.73 ± 3924.48	0.355
**Interleukin-6 (pg/ml)**	213.50 ± 133.94	253.86 ± 293.74	0.814
**Interleukin-10 (pg/ml)**	16.74 ± 12.03	10.00 ± 5.04	0.367
G test (pg/ml)			0.807
>37.5	2 (33.33%)	2 (50%)	
<37.5	4 (66.67%)	2 (50%)	
GM test (S/CO)			0.789
>0.25	2 (33.33%)	1 (25%)	
<0.25	4 (66.67%)	3 (75%)	
Pulmonary embolism			0.200
Yes	3 (50%)	0 (0%)	
No	3 (50%)	4 (100%)	

Normal data are represented as mean ± standard deviation; Non-normal data are represented as the median (lower quartile, upper quartile);.

Enumeration data are represented as the number of cases (proportion).

Measurement data conforming to normal distribution are expressed as mean ± standard deviation; skewed measurement data are expressed as median (interquartile range). Enumeration data are presented as n (%). mmHg, millimeters of mercury; ×10^9^/L, 10^9^ per liter; g/L, grams per liter; U/L, units per liter; ng/ml, nanograms per milliliter; pg/ml, picograms per milliliter; S/CO, optical density ratio of galactomannan assay. G test refers to 1,3-β-D-glucan assay; GM test refers to galactomannan assay.PTB: pulmonary tuberculosis subgroup, non-PTB: non-pulmonary tuberculosis subgroup; n represents the sample size of each group. P < 0.05 indicates statistically significant difference.

## 4 Discussion

Historically, the diagnosis of hematogenously disseminated tuberculosis has mainly relied on blood culture; however, this method has low sensitivity and a long culture period, and therefore cannot meet the clinical need for rapid diagnosis (Tyrrell et al., 2012). As an emerging culture-independent high-throughput sequencing technology, mNGS can detect a broad range of pathogens and offers the advantages of accuracy, comprehensiveness, and unbiased detection, with particularly prominent performance in detecting Mycobacterium tuberculosis and atypical pathogens (Simner et al., 2018). At present, mNGS is increasingly being applied in the diagnosis of tuberculosis and plays an indispensable role in the detection of Mycobacterium tuberculosis in pulmonary and extrapulmonary samples (Yan et al., 2020; Huang et al., 2023). For patients with suspected disseminated tuberculosis, blood mNGS testing is noninvasive, convenient, and associated with minimal contamination, making it an ideal option for diagnosing bloodstream infections (Peri et al., 2022). In this study, 10 HIV-negative patients with negative results on conventional etiologic testing were diagnosed with hematogenous disseminated tuberculosis by mNGS within 48 to 72 hours, suggesting that mNGS is an effective tool for the rapid diagnosis of this disease and can serve as a robust complement to traditional diagnostic methods.

Disseminated tuberculosis is more common in HIV-positive patients (Suárez et al., 2019). In this study, all patients were HIV negative; however, review of their medical histories showed that all patients had comorbid factors that could lead to impaired immune function (**Table 1**), thereby triggering disseminated tuberculosis (Crump and Reller, 2003; Salem, 2008; Sharma et al., 2016; Khan et al., 2016; Gaifer, 2017; Wang et al., 2022; Salvado et al., 2023). T cells are important immune cells in host defense against tuberculosis infection, and decreased CD4^+^ T-cell counts are associated with reduced lymphocyte counts, the occurrence of hematogenous disseminated tuberculosis, and patient death. In addition, worsening tuberculosis and an excessively high Mycobacterium tuberculosis load can also lead to insufficient T-cell numbers (Day et al., 2011) and even impair hematopoietic function (Li et al., 2023), forming a vicious cycle of immune injury (Li et al., 2020). The patients in this study also showed decreasing trends in lymphocyte and CD4^+^ T-cell counts, which is consistent with previous findings.

The clinical manifestations of disseminated tuberculosis are usually nonspecific, and symptoms vary depending on the organs and systems involved (Khan et al., 2016). The patients in this study mainly presented with general symptoms, especially nonspecific hyperpyrexia, and respiratory symptoms, which may be related to hematogenous dissemination of Mycobacterium tuberculosis and pulmonary involvement. Patients with disseminated tuberculosis may develop respiratory failure, and the mechanism is that the systemic inflammatory response induced by Mycobacterium tuberculosis damages the pulmonary vascular endothelium and alveolar epithelium, causing microthrombosis in small pulmonary vessels and intrapulmonary shunting (Kim et al., 2008). In this study, two-thirds of the patients developed mild to moderate respiratory failure, and the oxygenation index was significantly lower in the pulmonary tuberculosis subgroup than in the non-pulmonary tuberculosis subgroup. Theoretically, Mycobacterium tuberculosis can damage lung tissue or blood vessels through direct infiltration and induction of pulmonary immune responses, thereby aggravating oxygenation impairment (Shah and Reed, 2014). This suggests that patients with hematogenously disseminated tuberculosis complicated by pulmonary involvement may have poorer oxygenation function. However, it should be noted that, because of the small subgroup sample sizes (6 cases in the PTB subgroup and 4 cases in the non-PTB subgroup), this difference should be regarded as an exploratory finding and needs to be validated in prospective studies with larger sample sizes.

CRP is a nonspecific marker reflecting the level of systemic inflammation and is usually elevated during tuberculosis infection, with levels varying according to the site of infection and bacterial load (Brown et al., 2016). Studies have found that higher CRP concentrations (≥50 mg/L) are associated with the occurrence of disseminated tuberculosis and poor prognosis (Lawn et al., 2013). In this study, 80% of patients had CRP levels exceeding 50 mg/L, suggesting that markedly elevated CRP may indicate hematogenous disseminated tuberculosis. PCT levels in patients with tuberculosis are usually not significantly elevated, with 0.5 ng/mL used as the cutoff value (Ugajin et al., 2011). However, notably, more than half of the patients had PCT levels exceeding 0.5 ng/mL, which differs from the previous understanding that PCT is not markedly elevated in most patients with tuberculosis.A study by Huang et al. indicated that the elevated PCT group (PCT ≥0.5 ng/mL) was associated with disseminated tuberculosis (P = 0.007), and that PCT may serve as a biomarker of poor prognosis in pulmonary tuberculosis and disseminated tuberculosis, providing a basis for clinical risk stratification (Huang et al., 2014). In fact, PCT synthesis and release depend not only on the type and severity of infection but also on inflammatory cytokine cascades (Nyamande and Lalloo, 2006). Because the patients in this group had severe disease and some had coinfections, these observations require further confirmation in prospective studies.

Tuberculosis can cause vascular endothelial cells to produce large amounts of pro-inflammatory cytokines, such as IL-6 and TNF-α, leading to increased coagulation factors and placing the blood in a hypercoagulable state through regulation of antithrombin III, protein S, and protein C (Kager et al., 2015).Studies have shown that an increased risk of thrombosis is associated with the severity of tuberculosis (Suárez Ortega et al., 1993). In this study, elevated D-dimer levels and a relatively high proportion of thrombotic events were observed, and all 3 cases of pulmonary embolism occurred in the pulmonary tuberculosis subgroup; however, the difference did not reach statistical significance (P = 0.200). Given the limited sample size and the presence of pre-existing diseases in most patients, such as cardiovascular disease and diabetes mellitus, these findings should be regarded as preliminary observations and cannot yet be generalized to all HIV-negative patients with hematogenous disseminated tuberculosis. Therefore, a definitive conclusion regarding an association between pulmonary tuberculosis and pulmonary embolism cannot currently be drawn, and these findings only suggest that this direction warrants further attention in subsequent studies.

Chest imaging revealed pulmonary lesions in all patients in this study, with bilateral lung involvement in 90% of patients. Pulmonary tuberculosis was confirmed by etiological evidence in 6 cases, whereas Mycobacterium tuberculosis was not detected in pulmonary samples from the remaining 4 cases. Unlike the classic CT findings of hematogenous disseminated tuberculosis, which are characterized by diffuse miliary nodules in the lungs, the imaging manifestations in the 10 HIV-negative patients mainly included patchy ground-glass opacities, consolidation, diffuse lung nodules, pleural effusion, and pericardial effusion, most of which lacked diagnostic specificity.

In this study, 2 patients with tuberculous pleurisy confirmed by mNGS had bilateral pleural effusion, a finding inconsistent with typical tuberculous pleural effusion. This suggests that the possibility of tuberculous pleurisy cannot be completely excluded even in cases of atypical pleural effusion. When bilateral pleural effusion cannot be explained by other causes, mNGS may be used for etiologic screening and disease assessment. In fact, the imaging manifestations of tuberculosis largely depend on patients’ immune status (Wetscherek et al., 2022), and the advantages of mNGS are more pronounced in patients with impaired immune function and atypical imaging findings.

This study has several limitations. First, the sample size was small. Second, some patients had coinfections with other pathogens, which may have affected certain symptoms and interfered with clinical judgment. In a sense, however, this also highlights the advantages of mNGS, which does not require specific amplification and can rapidly and objectively identify pathogenic microorganisms in clinical specimens through a single test.

## Data Availability

The original contributions presented in the study are included in the article/supplementary material. Further inquiries can be directed to the corresponding author.
